# Development and characterization of EST‐SSR markers for *Vitex negundo* var. *heterophylla* (Lamiaceae)

**DOI:** 10.1002/aps3.1209

**Published:** 2019-01-07

**Authors:** Lele Liu, Jingwen Wang, Meiqi Yin, Xiao Guo, Yunfei Cai, Ning Du, Xiaona Yu, Weihua Guo

**Affiliations:** ^1^ Institute of Ecology and Biodiversity School of Life Sciences Shandong University Qingdao 266237 People's Republic of China; ^2^ College of Landscape Architecture and Forestry Qingdao Agricultural University Qingdao 266109 People's Republic of China; ^3^ School of Life Science Qilu Normal University Jinan 250100 People's Republic of China

**Keywords:** expressed sequence tag–simple sequence repeat (EST‐SSR) markers, Lamiaceae, transcriptome sequencing, *Vitex negundo* var. *heterophylla*, *Vitex rotundifolia*

## Abstract

**Premise of the Study:**

*Vitex negundo* var. *heterophylla* (Lamiaceae) is a dominant shrub in the warm temperate zone of northern China. Expressed sequence tag–simple sequence repeat (EST‐SSR) markers were developed to investigate its genetic diversity and structure.

**Methods and Results:**

We detected 12,075 SSRs in *V. negundo* var. *heterophylla* using transcriptome sequencing. Primer pairs for 100 SSR loci were designed and amplified in three populations of *V. negundo* var. *heterophylla*. Sixty loci were amplified, of which 14 were polymorphic. The number of alleles per locus ranged from two to 15, and levels of observed and expected heterozygosity ranged from 0.241 to 0.828 and from 0.426 to 0.873, respectively. All primer pairs amplified PCR products from *V. rotundifolia* but only four of them amplified products from *Leonurus japonicus*.

**Conclusions:**

The identified EST‐SSR markers will be useful for future molecular and reproductive ecology studies of *V. negundo* var. *heterophylla* and *V. rotundifolia*.

*Vitex negundo* L. var. *heterophylla* (Franch.) Rehder (Lamiaceae) is a deciduous shrub species that is widely distributed in the hilly areas of northern China. It exhibits a range of morphological and physiological adaptations to abiotic environmental factors, such as water and light regimes (Du et al., 2010, 2012, 2017), that are under the control of genetic and epigenetic mechanisms (Liu et al., 2018). This has allowed colonization of a broad range of habitats, including woodland, bush, and roadsides. Only a few genetic studies of *V. negundo* have been reported, and these have been based on random amplified polymorphic DNA (RAPD) (Su et al., 2003; Zhang et al., 2007) and amplified fragment length polymorphism (AFLP) markers (Liu et al., 2018). Notably, no studies using codominant genetic markers are currently available. In *V. rotundifolia* L. f. (Lamiaceae), an endangered coastal species, genomic simple sequence repeat (gSSR) markers have been reported (Ohtsuki et al., 2014). Here, we sequenced the transcriptome and developed expressed sequence tag–SSR (EST‐SSR) markers for *V. negundo* var. *heterophylla*. These markers will be useful for further reproductive and evolutionary ecology studies of *V. negundo* var. *heterophylla* and can also provide information for revegetation and management programs.

## METHODS AND RESULTS

Two *V. negundo* var. *heterophylla* individuals were sampled for transcriptome sequencing from Fohui Mountain in Jinan, Shandong Province, China (Appendix 1). RNA was extracted from collected leaves using the RNAprep Pure Plant kit (Tiangen, Beijing, China), and mRNA was isolated from total RNA using the NEBNext Ultra RNA Library Prep Kit (New England Biolabs, Ipswich, Massachusetts, USA). After ultrasonic fragmentation, mRNA was converted to double‐stranded cDNA using the same kit. Purification and size selection were conducted using AMPure XP Beads (Beckman Coulter, Brea, California, USA). Finally, DNA fragments of approximately 400 bp in length were sequenced using an Illumina HiSeq instrument (Illumina, San Diego, California, USA). The raw data were deposited in the National Center for Biotechnology Information (NCBI) Sequence Read Archive (accession no. PRJNA491662). The raw sequences were filtered by removing adapters and low‐quality reads (quality score < 30), resulting in 45.568 and 43.082 million clean reads, from the two libraries, respectively. These reads were de novo assembled using Trinity (Grabherr et al., 2011) into 52,072 unigenes, with an N50 length of 1414 bp. The putative functions of EST‐SSR sequences were determined by BLASTX against the NCBI non‐redundant protein (nr) database. We detected 12,075 SSR loci from these unigenes using MISA (Thiel et al., 2003), consisting of 4242 mononucleotide SSRs and 7833 di‐, tri‐, tetra‐, penta‐, and hexanucleotide SSRs. Primers were designed using Primer3 (Untergasser et al., 2012).

Fresh leaves were collected from three populations of *V. negundo* var. *heterophylla* in Shandong Province, China (Appendix 1), and genomic DNA was extracted from dried leaf tissue using the cetyltrimethylammonium bromide (CTAB) method (Doyle and Doyle, 1987). Initially, we used 100 primer pairs with high dinucleotide or trinucleotide repeat motifs to amplify products from six individuals belonging to three populations. PCR amplification was performed in a final volume of 20 μL, containing 3 ng of template DNA, 2 μL of 10× buffer (with Mg^2+^; Tiangen), 1 μL of dNTPs (2.5 mM each), 1 μL of each primer (5 μM), and 1 unit of *Taq* polymerase (Tiangen). The PCR program consisted of an initial denaturation step at 95°C for 5 min; followed by 35 cycles of denaturation at 95°C for 30 s, annealing at an appropriate temperature for 1 min, and extension at 72°C for 45 s; followed by a final extension step at 72°C for 7 min and 65°C for 30 s. The PCR products were fractionated by electrophoresis using both 2% agarose gels and 6% polyacrylamide gels with a 1‐kbp DNA Ladder Marker (Tiangen) as a reference. In total, 52 primer pairs amplified detectable products, of which 14 pairs showed polymorphism among the six tested samples (Table 1; see monomorphic loci in Appendix 2). The putative functions of EST‐SSR sequences were determined by BLASTX against the NCBI nr database.

**Table 1:** Characteristics of 14 polymorphic EST‐SSR markers developed for *Vitex negundo* var. *heterophylla*

Locus	Primer sequences (5′–3′)	Repeat motif	*T*_a_ (°C)	Expected allele size (bp)	Allele size range (bp)	Putative function [Organism]	*E*‐value	GenBank accession no.
V02	F: AGCAGGGAGAGGAAGAGGAG	(TGG)_13_	58	172	156–187	No hit	—	MH825839
	R: ACCAACCCCACTCAGCTAGA							
V07	F: CCTCTGCTGCGCATGTCTAT	(AG)_16_	56	125	107–133	Serine hydroxymethyltransferase, mitochondrial [*Erythranthe guttatus*]	8.90E‐114	MH825840
	R: TAAGGGGCTTGCCAATGGAG							
V15	F: CAACAGAGAGGGCGTCAAGT	(AT)_6_	56	220	206–237	No hit	—	MH825841
	R: GGGGAGTGTCGAAGTGGAAG							
V25	F: ACAGCAGCCATTCAGACTGT	(GT)_16_	58	236	207–253	No hit	—	MH825842
	R: CGTTGCATTCGGCCATTCAA							
V30	F: GCAAGGCGAAGAATACAGCG	(TGC)_5_	56	191	189–205	ABC transporter G family member 11 [*Sesamum indicum*]	6.20E‐172	MH825843
	G: GTCGGGAGGGACTGAGTAGT							
V49	F: CCGTTCGCTGTTGCTTGTAC	(AG)_14_	56	215	200–242	No hit	—	MH825844
	R: CCTCAGCAGTTTGGACGTCT							
V55	F: GCAAGCTCCTCCTTCCTTGA	(CTC)_10_	56	198	276–206	Probable protein *S*‐acyltransferase 12 isoform X3 [*Sesamum indicum*]	6.90E‐99	MH825845
	R: ACCGAGGAAGTTGAGTGCAG							
V59	F: AGCTGAATGGCAACCTTCGA	(GAT)_7_	56	238	224–237	Intracellular protein transport protein USO1‐like [*Sesamum indicum*]	8.80E‐78	MH825846
	R: ACGAGGTCCTCTAGTGCCTT							
V70	F: TGTTGGCCGATCAGCTGATT	(GCT)_7_	56	144	133–153	Hypothetical protein MIMGU_mgv1a000263mg [*Erythranthe guttata*]	1.00E‐146	MH825847
	R: GCAGCAGCCTTCCATTATGC							
V76	F: TGACGCTCTCGATCCAACTT	(AG)_15_	56	121	94–128	Uncharacterized protein At4g26450 [*Sesamum indicum*]	5.50E‐52	MH825848
	R: GCCTTGGCCATCATTTCAGC							
V95	F: CGAGTATACGCAGGCGAACT	(GCC)_7_	56	253	242–266	Zinc finger protein 8 [*Erythranthe guttatus*]	6.10E‐13	MH825849
	R: GCTTGGCTGATGCACATGTT							
V97	F: GTCACCACTCACCGGCAATA	(CA)_12_	56	229	211–239	Uncharacterized protein LOC105175071 [*Sesamum indicum*]	2.90E‐40	MH825850
	R: GGCGCGTCATGGTATAAGGA							
V99	F: ACGACGAGCTCGAACATGAA	(GTG)_8_	56	161	155–173	Transcription factor bHLH63 [*Sesamum indicum*]	5.50E‐52	MH825851
	R: GATACGCAGCAGCAGAGGAT							
V100	F: CTGCCACCACCTCCATTTCT	(CAA)_8_	56	220	211–235	d‐3‐phosphoglycerate dehydrogenase 2, chloroplastic‐like [*Sesamum indicum*]	1.20E‐293	MH825852
	R: TCGGAATCCTTCACCAGCAC							

Note

*T*_a_ = annealing temperature.

The 14 primer pairs were then used with all 83 samples from the three populations to evaluate the overall level of polymorphism. The forward primers were 5′ end‐labeled with FAM dye, and final products were fractionated using an ABI 3730XL DNA capillary sequencer (Applied Biosystems, Foster City, California, USA) with a LIZ 500 Internal Size standard (Applied Biosystems). GenAlEx version 6.5 (Peakall and Smouse, 2012) was used to calculate the number of alleles, observed heterozygosity, and expected heterozygosity for each locus. GENEPOP software (version 4.7.0; Rousset, 2008) was used to investigate linkage disequilibrium and to determine deviation from Hardy–Weinberg equilibrium. The number of alleles per locus ranged from two to 15, the levels of observed heterozygosity ranged from 0.241 to 0.828, and the levels of expected heterozygosity ranged from 0.426 to 0.873 (Table 2). Significant linkage disequilibrium was detected between loci V15 and V30 (*P* = 0.0109) and loci V25 and V70 (*P* = 0.0266). Loci V97 and V100 showed significant deviation from Hardy–Weinberg equilibrium in two populations (*P* < 0.001; Table 2).

**Table 2:** Genetic variation in the 14 polymorphic EST‐SSR markers in three *Vitex negundo* var. *heterophylla* populations.*

Locus	Population A (*n* = 29)			Population B (*n* = 28)			Population C (*n* = 26)		
	*A*	*H*_o_	*H*_e_	*A*	*H*_o_	*H*_e_	*A*	*H*_o_	*H*_e_
V02	7	0.655	0.746	8	0.786	0.815	9	0.692	0.844
V07	7	0.759	0.804	7	0.714	0.751	8	0.692	0.834
V15	7	0.536	0.573	8	0.500	0.670	6	0.615	0.698
V25	15	0.828	0.873	12	0.714	0.865	13	0.692	0.719
V30	4	0.655	0.624	5	0.500	0.466	5	0.577	0.679
V49	10	0.586	0.757	10	0.714	0.836	11	0.538	0.771
V55	7	0.759	0.687	9	0.714	0.733	7	0.538	0.562
V59	3	0.621	0.540	3	0.500	0.517	3	0.538	0.514
V70	6	0.828	0.719	8	0.714	0.746	6	0.731	0.724
V76	12	0.690	0.699	11	0.786	0.811	8	0.615	0.754
V95	6	0.379	0.479	7	0.643	0.629	10	0.500	0.509
V97	8	0.345	0.717^a^	9	0.393	0.790^a^	8	0.385	0.798
V99	4	0.241	0.456	5	0.536	0.573	2	0.385	0.426
V100	8	0.724	0.744^a^	6	0.357	0.651^a^	8	0.500	0.743

Note

*A* = number of alleles; *H*_e_ = expected heterozygosity; *H*_o_ = observed heterozygosity; *n* = sample size.

* Locality and voucher information are provided in Appendix 1.

a Significant deviation from Hardy–Weinberg equilibrium (*P* < 0.001).

To test the transferability of the 14 primers between taxa, they were used with DNA samples from *V. rotundifolia* and *Leonurus japonicus* Houtt. (Lamiaceae). All primer pairs successfully amplified products from *V. rotundifolia*, but only four primer pairs amplified products from some *L. japonicus* individuals (Table 3).

**Table 3:** Cross‐amplification of 14 polymorphic EST‐SSR markers developed for *Vitex negundo* var. *heterophylla* in *V. rotundifolia* and *Leonurus japonicus*.^a^

Locus	*Vitex rotundifolia* (*n* = 13)^b^	*Leonurus japonicus* (*n* = 8)
V02	169, 172, 175, 187	—
V07	117, 123	—
V15	208, 233*	—
V25	225, 227, 229*	*
V30	191, 193, 195	—
V49	200, 210, 214	—
V55	176, 179, 185	—
V59	224, 234, 237	—
V70	140, 149	—
V76	110, 112, 122, 128	*
V95	245, 248, 254	*
V97	211, 221, 223, 227*	*
V99	167, 170, 173	—
V100	211, 214, 217, 220, 226, 229	—

Note

* = primers amplified products in some individuals; — = primers did not amplify in any of the individuals; *n* = number of individuals.

a Locality and voucher information are provided in Appendix 1.

b Numbers represent the PCR product size.

## CONCLUSIONS

We assembled 52,072 unigenes of *V. negundo* var. *heterophylla* following transcriptome sequencing and used this data set to develop 14 novel polymorphic EST‐SSR primer pairs. All of these primers amplified products in the related species *V. rotundifolia*. These markers represent a useful resource for reproductive and genetic ecology studies of this species and may provide a valuable tool for revegetation and management in northern China.

## AUTHOR CONTRIBUTIONS

W.G., L.L., and Y.C. conceived and designed the experiments. L.L., J.W., M.Y., X.G., X.Y., and N.D. contributed to sample collection. L.L. and J.W. performed the molecular laboratory work. L.L., J.W., M.Y., and Y.C. participated in data pre‐processing. L.L., Y.C., N.D., X.Y., and W.G. analyzed the data. L.L. drafted the manuscript and all authors participated in manuscript modifications and gave final approval for publication.

## DATA ACCESSIBILITY

All sequence information was uploaded to the National Center for Biotechnology Information (NCBI) Sequence Read Archive (accession no. PRJNA491662); primer sequences were uploaded to GenBank (accession no. MH825839–MH825852 and MH892533–MH892570; Table 1 and Appendix 2).

## APPENDIX 1 Voucher information for *Vitex negundo* var. *heterophylla, V. rotundifolia*, and *Leonurus japonicus* individuals used in this study.

**Table:**

Species	Population	*N*	Collection locality^a^	Geographic coordinates	Voucher specimen^b^
*Vitex negundo* L. var. *heterophylla* (Franch.) Rehder	A	29	Fanggan	36.4317°N, 117.4516°E	01611001
*Vitex negundo* var. *heterophylla*	B	28	Mengshan	35.5376°N, 117.9895°E	01611002
*Vitex negundo* var. *heterophylla*	C	26	Yaoxiang	36.3213°N, 117.1200°E	01611003
*Vitex negundo* var. *heterophylla*	—	2	Jinan	36.6317°N, 117.0347°E	01709001^c^01709002^d^
*Vitex rotundifolia* L. f.	—	13	Muping	37.4574°N, 121.6826°E	01801001
*Leonurus japonicus* Houtt.	—	8	Jinan	36.7239°N, 117.0207°E	01801002

a Collection localities are in Shandong Province, China.

b All voucher specimens were collected by Lelel Liu and are deposited in the Institute of Ecology and Biodiversity, School of Life Sciences, Shandong University (JSPC), Qingdao, China. The sample with irregularly pinnatifid leaflets (^c^) and the sample with slightly incised leaflets (^d^) were used for transcriptome sequencing.

## APPENDIX 2 Characteristics of the 38 monomorphic EST‐SSR markers developed for *Vitex negundo* var. *heterophylla*.

**Table:**

Locus	Primer sequences (5′–3′)	Repeat motif	*T*_a_ (°C)	Allele size (bp)	Putative function [Organism]	*E*‐value	GenBank accession no.
V3	F: CGATGATGCCCCCACTAGTC	(TC)_15_	56	150	Unnamed protein product [*Coffea canephora*]	2.80E‐17	MH892533
	R: TCCGCAGATGGCCTGTTATC						
V4	F: TCTCTCTTCTCTCCTCCGCC	(CAA)_6_	56	207	Uncharacterized protein LOC105175754 [*Sesamum indicum*]	1.50E‐60	MH892534
	R: GGGTCTTCGGAAATGGGGTT						
V8	F: ACGCGAACCTGTGAAGATGT	(CT)_12_	56	171	Hypothetical protein M569_03371, partial [*Genlisea aurea*]	2.50E‐56	MH892535
	R: GAAACAAGGAAGCGACGCTC						
V11	F: TGATGCCATGGTAGCAACGA	(AAG)_9_	56	248	G‐type lectin S‐receptor‐like serine/threonine‐protein kinase At4g27290 [*Sesamum indicum*]	1.20E‐15	MH892536
	R: GTTCGAACTTCCCACCGGAT						
V13	F: TAAGACTCCCACTGCAAGCG	(CCA)_5_	56	212	Uncharacterized protein LOC105974765 [*Erythranthe guttatus*]	3.20E‐50	MH892537
	R: GAATGTGGCAGGTGGATCCA						
V18	F: GGAACACGTGATTGGGGTTC	(TC)_16_	56	193	No hit	—	MH892538
	R: AGACGGGCGAAAAACTCCAA						
V21	F: CCGGAAAAAGCAGTAACCGC	(CT)_13_	56	209	Unnamed protein product [*Vitis vinifera*]	5.10E‐06	MH892539
	R: ATCACCAGCAACTGCCATCA						
V26	F: CAGCAGCCCCAAATTTGCAA	(GGC)_8_	56	241	26S proteasome subunit RPT2B [*Arabidopsis thaliana*]	5.10E‐06	MH892540
	R: GAGCTGGTCCTAATCGGCAA						
V27	F: AGTCTGTGCCTTGTTGCTGA	(TG)_11_	56	243	Uncharacterized protein LOC105158430 [*Sesamum indicum*]	2.40E‐65	MH892541
	R: ACTTTGCACCCCTCAATCCA						
V33	F: GACGTCCCCATTCGGAACTC	(CT)_17_	56	258	No hit	—	MH892542
	R: GCTTCTCCACTCGACTGTCA						
V36	F: TACGCCTATGTTTGTGGCCA	(AG)_10_	56	182	Pentatricopeptide repeat‐containing protein At5g67570, chloroplastic [*Sesamum indicum*]	7.00E‐118	MH892543
	R: TTATGAGCTAGCTCGCTGCC						
V41	F: CGGCCGGAGCAAGAAGATAT	(GA)_10_	56	245	Myosin‐14 [*Sesamum indicum*]	2.90E‐127	MH892544
	R: CTCTCTTGCCGGAGCTTCAT						
V43	F: AGCAAGCCGGAATGAATCGA	(AAC)_7_	56	221	Transcription factor GTE7‐like [*Sesamum indicum*]	1.50E‐111	MH892545
	R: TGGACGTCTGGTTGAACGAG						
V47	F: TGGAAGCCTGTGTTGTGTGA	(GA)_17_	56	152	Haloacid dehalogenase‐like hydrolase domain‐containing protein SGPP [*Erythranthe guttatus*]	9.90E‐75	MH892546
	R: AGTTCCGTCAAGCGAGGAAG						
V48	F: CCACAAATGCAGCGAGTTCA	(CAG)_8_	56	103	Unnamed protein product [*Coffea canephora*]	9.90E‐198	MH892547
	R: TTCCAGATGCAGGCTGTAGC						
V50	F: CCACTAATCGCAACAGCAGC	(TC)_13_	56	258	Phospholipase SGR2‐like isoform X1 [*Sesamum indicum*]	3.60E‐187	MH892548
	R: GGTAGCACATGGCCATCAGT						
V51	F: CCGGTTTGGAGTTTGCCTTG	(GGC)_7_	56	165	Uncharacterized protein LOC105172005 isoform X1 [*Sesamum indicum*]	2.10E‐83	MH892549
	R: AGCACACAGATCACCGATGG						
V52	F: CCAGCGCAAGACGTACTACT	(AG)_38_	56	260	Uncharacterized protein LOC105171026 [*Sesamum indicum*]	1.30E‐36	MH892550
	R: CTCTCAGCTCGTTGGCAGAA						
V53	F: AACACCGGCGAGTTGAGTAG	(AG)_14_	56	115	Hypothetical protein POPTR_0007s12520g [*Populus trichocarpa*]	7.10E‐33	MH892551
	R: ACAGTCACAGTGTGGCACAT						
V54	F: CGCCTCTCACAGTCATACCG	(TG)_15_	56	149	No hit	—	MH892552
	R: CTCAAGTCTCAGCCACGCA						
V56	F: ACCATTTGCTTCGCATACGC	(GGA)_7_	56	135	No hit	—	MH892553
	R: CACATGGTCGAAGCCTAGCA						
V58	F: AAGCTGCTGCCACCATTGTA	(GTT)_7_	56	269	Protein E6‐like [*Sesamum indicum*]	2.00E‐24	MH892554
	R: AACAGCTACGGCCTTTACGG						
V61	F: GGCTCAGAAGGCCAAGACAT	(GA)_11_	56	159	Galacturonokinase [*Sesamum indicum*]	5.10E‐165	MH892555
	R: TCTTCAACGCAACTCCACCA						
V63	F: CCATGACGTCGGAGGAGATG	(AG)_11_	56	275	Uncharacterized protein LOC105970868 [*Erythranthe guttatus*]	8.20E‐51	MH892556
	R: TCTCGTCCAAACACGCCATT						
V64	F: ACGACCTGGATTTCGACCAC	(AG)_11_	56	168	No hit	—	MH892557
	R: GCACGCACACACAACACAAT						
V66	F: TCTTGATCAGCTGCCACCAG	(TCA)_8_	56	234	Uncharacterized protein LOC105157368 [*Sesamum indicum*]	1.10E‐31	MH892558
	R: GAGCTTGGTTAGTGGCGAGA						
V71	F: CACTCCGACCACTTGAAGCT	(TC)_13_	56	162	Protein IQ‐DOMAIN 32‐like [*Sesamum indicum*]	5.90E‐60	MH892559
	R: GTGAAGCGAGGAGACCAACA						
V72	F: TCAAGCGGCTCGTATGAGTC	(TC)_13_	56	123	Uncharacterized protein LOC105170218 [*Sesamum indicum*]	1.30E‐74	MH892560
	R: CATCACCGGCGAAACAACTG						
V82	F: GCAAGAGCCTAGTCGAGCTT	(ATG)_9_	56	248	RNA exonuclease 3 [*Gossypium arboreum*]	1.90E‐22	MH892561
	R: AGTCCATGCCTCCGACAAAT						
V83	F: TCCACCACCACTCAAAGACG	(CT)_17_	56	139	Protein GAR2 isoform X1 [*Sesamum indicum*]	7.90E‐98	MH892562
	R: CCTGCCAACTCTCATTCCGT						
V84	F: CAGTGAAGAGCGCAGGAAGA	(GCG)_8_	56	183	Uncharacterized protein LOC105176253 isoform X1 [*Sesamum indicum*]	2.00E‐253	MH892563
	R: CCTCCTCTCGCTTCCATCAC						
V88	F: TTGGTCCTGCAAGCATAGCA	(AG)_23_	56	189	No hit	—	MH892564
	R: TGCCAACCGGTTCTAAGTCA						
V89	F: TCGCGTAGTCCAGCTTCTTC	(AG)_11_	56	149	Reactive oxygen species modulator 1‐like [*Sesamum indicum*]	1.90E‐20	MH892565
	R: ATAAACAGCACCAACAGCGC						
V90	F: ACGAGTCGCCATTGTCGATT	(GT)_16_	56	151	Probable leucine‐rich repeat receptor‐like protein kinase At1g35710 [*Sesamum indicum*]	1.60E‐185	MH892566
	R: CGTCTCCAACTCGACTGCTT						
V92	F: GGAAATCAGTTGCCTTGCCG	(AGC)_7_	56	186	E3 ubiquitin‐protein ligase RGLG2‐like, partial [*Pyrus ×bretschneideri*]	1.60E‐87	MH892567
	R: GCAAGTCATGTGTCCACAGC						
V93	F: CAAGTAATCGCCGTGAACCG	(CGC)_7_	56	251	Uncharacterized protein LOC105173283 [*Sesamum indicum*]	6.30E‐51	MH892568
	R: ACTTCACTCTGCCGCATCTC						
V94	F: CGGAGAAAGCCATGCACATG	(GAA)_8_	56	226	Hypothetical protein MIMGU_MGV1A020013MG [*Erythranthe guttata*]	1.90E‐18	MH892569
	R: TCGTATCAGGAGCAGAGCCA						
V96	F: AGGCACGAAAGCAAGAGTGT	(GGC)_7_	56	177	Uncharacterized protein LOC105968457 [*Erythranthe guttatus*]	3.80E‐40	MH892570
	R: GAGTCGCCTCCTCCAATCTG						

Note

*T*_a_ = annealing temperature.
